# The stability-plasticity dilemma: investigating the continuum from catastrophic forgetting to age-limited learning effects

**DOI:** 10.3389/fpsyg.2013.00504

**Published:** 2013-08-05

**Authors:** Martial Mermillod, Aurélia Bugaiska, Patrick Bonin

**Affiliations:** ^1^Centre National de la Recherche Scientifique, LPNC UMR 5105, Université Grenoble AlpesGrenoble, France; ^2^Institut Universitaire de FranceParis, France; ^3^LEAD-Centre National de la Recherche Scientifique, UMR 5022, University of BourgogneDijon, France

The stability-plasticity dilemma is a well-know constraint for artificial and biological neural systems. The basic idea is that learning in a parallel and distributed system requires plasticity for the integration of new knowledge, but also stability in order to prevent the forgetting of previous knowledge. Too much plasticity will result in previously encoded data being constantly forgotten, whereas too much stability will impede the efficient coding of this data at the level of the synapses. However, for the most part, neural computation has addressed the problems related to excessive plasticity or excessive stability as two different fields in the literature.

## The problem of catastrophic forgetting for distributed neural networks

The problem of catastrophic forgetting has emerged as one of the main problems facing artificial neural networks. The problem can be stated as follow: a distributed neural system, for example any biological or artificial memory, has to learn new inputs from the environment but without being disrupted by them. Catastrophic forgetting is defined as a complete forgetting of previously learned information by a neural network exposed to new information (McCloskey and Cohen, 1989; Ratcliff, 1990). This problem is a general problem that exists in different types of neural networks from standard back-propagation neural networks to unsupervised neural networks like self-organizing maps (Richardson and Thomas, 2008) or for connectionist models of sequence acquisition (Ans et al., 2002). Concerning artificial connectionist neural networks (such as, for instance, standard backpropagation networks), they are highly sensitive to catastrophic forgetting because of their highly distributed internal representations (French, 1992). Therefore, it is possible to reduce the problem of catastrophic forgetting by reducing the overlap among the internal representations stored in the neural network, for example using larger systems, or for example sparse or interleaved learning (Hetherington and Seidenberg, 1989; McRae and Hetherington, 1993). For this reason, when learning input patterns, connectionist networks have to alternate between them and adjust the corresponding synaptic weights by small increments in order to appropriately associate each input vector with the related output vector. By contrast, sequential learning in a standard connectionist network would result in the complete forgetting of previously learned input-output patterns. This problem affecting artificial neural networks clearly distinguishes them from the cognitive abilities of biological neural systems that are able to learn new patterns in sequential order without catastrophic forgetting.

In order to prevent catastrophic forgetting, various researchers have suggested using a dual-memory system which, fundamentally, simulates the presence of a short-term and a long-term memory (Robins, 1995; Ans and Rousset, 1997; French, 1997; Mermillod et al., 2003). The principle is to consolidate information, initially present in a short-term memory, within a long-term memory in order to prevent catastrophic forgetting in connectionist systems. This principle, investigated within the perspective of neural computation in artificial systems, could also point the way to a more general principle that also applies to biological neural systems (French, 1999).

## The entrenchment effect: the opposite extreme of the plasticity-stability dilemma

At the opposite extreme of the stability-plasticity continuum lies the entrenchment effect, which may contribute to age-limited learning effects (Zevin and Seidenberg, 2002; Bonin et al., 2004, 2009; Mermillod et al., 2009a). In the cognitive sciences, this research field emerged as part of the attempt to determine whether items which are acquired early in life are better memorized in adults than those which are acquired later in life. Various studies working within this perspective have shown that words acquired early are processed faster and more accurately than words acquired later in life (see Juhasz, 2005; Johnston and Barry, 2006 for reviews). These so-called age-of-acquisition effects have been found in a large variety of tasks, for example picture naming tasks, as well as in different populations (e.g., children and adults).

While distributed neural networks have long been used to address various issues in word recognition and spoken word production studies, they have also recently been used to investigate the computational basis of these age-limited learning effects (e.g., Ellis and Lambon Ralph, 2000; Zevin and Seidenberg, 2002; Lambon Ralph and Ehsan, 2006). In these connectionist models, lexical frequency is encoded in the strength of the connections between the different types of representations which are involved in recognizing and producing words (Seidenberg and McClelland, 1989; Plaut et al., 1996). As far as connectionist simulations of age-limited learning effects are concerned, Ellis and Lambon Ralph (2000) were the first to show that the order of introduction of the encounters determines the number of errors produced by the neural network at the end of training. More precisely, the items introduced first in their study produced fewer errors than the late-introduced items, even after cumulative frequency had been carefully controlled for. This effect of age-limited learning effects in connectionist networks is referred to as the entrenchment effect.

At a computational level, the question is to understand how this entrenchment effect emerges. According to Zevin and Seidenberg (2002), the loss of plasticity in connectionist networks such as Seidenberg and McClelland's (1989) was due to the adjustments of the weights that occur on the basis of the logistic function used by the backpropagation algorithm and permits adjustments to the weights (initially set to random values between 0 and 1). These adjustments are at their largest when the activations occur in the middle of the logistic function (around 0.5) and become smaller as the weights converge on values that cause unit activations to approximate more closely to the target values (for instance 1 or 0). Thus, there is a loss of plasticity (early trained patterns become entrenched in the weights) associated with the learning of the first patterns in the training regime. Therefore, according to Zevin and Seidenberg (2002), the loss of plasticity in connectionist systems should vary as a function of the transfer function and the error signal computed. For example, a root mean square vs. cross-entropy error should produce different sensitivity to the entrenchment effect, but also to catastrophic forgetting. Of course, other factors as competition effects, loss of resources, and assimilation effects are important to produce age limited learning effects (Thomas and Johnson, 2006) and are important to control as possible confounded variables. In the current article, we suggest that the Fahlman offset (Fahlman, 1988) could constitute a simple and efficient way to test the computational basis of the loss of plasticity assumed by Zevin and Seidenberg (2002).

## The fahlman offset: a way to investigate both ends of the continuum

It is interesting to note that the above-mentioned research fields investigate two extremes of the same continuum. In other words, the entrenchment effect is related to a lack of plasticity (and an excess of stability) in response to newly acquired items, whereas catastrophic interference is related to an excess of plasticity (and a lack of stability) in response to new items presented sequentially. There are a number of ways of overcoming this difficulty, for instance by manipulating the orthogonality or the sparseness of the input-output patterns (French, 1992; Robins, 1995). However, among the different possibilities proposed to modulate the plasticity of neural networks, the method proposed by Fahlman (1988) is both simple and efficient. The basic idea is to add a constant number to the derivative of the sigmoid function (synaptic weights are adjusted by multiplying the error produced by a neuron by the derivative of the transfer function, i.e., the sigmoid function). This method makes it possible to avoid the entrenchment effect in the flat part of the sigmoid function and is relevant because this entrenchment effect is due to the flat spots at which the derivative of the sigmoid function approaches zero. Once the output value of a trained neural network starts to become entrenched around this flat spot of the sigmoid function, it becomes very difficult for the standard backpropagation algorithm to modify the synaptic weights responsible for producing this error. Even if an output value represents the maximum possible error, a unit whose output is close to 0.0 or 1.0 will be able to backpropagate only a tiny fraction of this error to the incoming weights and to units in earlier layers. Although it is theoretically possible to recover from entrenchment, this takes a very long time. The method proposed by Fahlman (1988), which consists of adding a small constant number to the derivative of the sigmoid function so that it does not go to zero for any output value, is therefore, both very simple and efficient to improve the efficiency of connectionist networks to simulate human cognitive processes (Mermillod et al., 2009b, 2010). For example, adding a constant of 0.1 to the sigmoid function before using it to scale the error prevents neuron values from approaching 0 and avoids the flat spots in the sigmoid function where the synaptic weights can become entrenched.

## New findings and perspective

In a recent article (Mermillod et al., 2012), we showed that age of acquisition can be considered, at a computational level, as an extreme case of frequency trajectory (i.e., the frequency with which a word is encountered during a certain period of life) and can help explain age-limited learning effects. Interestingly, no age-limited learning effects appeared when we used a Fahlman offset of 0.1 whereas it reappeared when we used a Fahlman offset of 0.0. This result was not consistent with Ellis and Lambon Ralph (2000) who reported an age-limited learning effect despite the improvement in the plasticity of the neural network brought about by modulating the Fahlman offset. This could be due to differences in the number or size of the training set between the two studies (Ellis and Lambon Ralph, 2000 or Mermillod et al., 2012). Therefore, the role of the training set in modulating the effects of learning parameters on age-limited learning and catastrophic interference remains a target of further investigation (since these factors could have a combined effect with neural plasticity). However, our results were not unambiguous: modifying the plasticity of an identical neural network by manipulating the Fahlman offset clearly modified the ability of the neural network to simulate (or not) age-limited learning effects. On the other side of the continuum, when the Fahlman constant was set to 0.0, we observed the age-limited learning effects reported in the literature (Ellis and Lambon Ralph, 2000; Zevin and Seidenberg, 2002; Lambon Ralph and Ehsan, 2006). Moreover, one result that will surprise researchers working in the field of catastrophic forgetting is that this catastrophic forgetting effect was largely reduced after the period of entrenchment of synaptic weights (early acquired patterns for “adult” networks having been learnt at an early stage, compared to the medium and late patterns being learnt sequentially in a later stage). To conclude, we suggest here that investigating the plasticity-stability continuum by modulating the Fahlman offset should help us understand a wide range of cognitive phenomena from age-limited learning effects through to catastrophic forgetting, as well as various forms of memory disorders.
